# 1,5-Dimethyl-3-propargyl-1*H*-1,5-benzodiazepine-2,4(3*H*,5*H*)-dione

**DOI:** 10.1107/S1600536810024219

**Published:** 2010-06-26

**Authors:** Rachid Dardouri, F. Ouazzani Chahdi, Natalie Saffon, El Mokhtar Essassi, Seik Weng Ng

**Affiliations:** a Laboratoire de Chimie Organique Appliquée, Faculté des Sciences et Techniques, Université Sidi Mohamed Ben Abdallah, Fés, Morocco; b Service Commun Rayons-X FR2599, Université Paul Sabatier, Bâtiment 2R1, 118 route de Narbonne, Toulouse, France; c Laboratoire de Chimie Organique Hétérocyclique, Pôle de Compétences Pharmacochimie, Université Mohammed V-Agdal, BP 1014 Avenue Ibn Batout, Rabat, Morocco; d Department of Chemistry, University of Malaya, 50603 Kuala Lumpur, Malaysia

## Abstract

The asymmetric unit of the title compound, C_14_H_14_N_2_O_2_, comprises two independent mol­ecules, which slightly differ in the orientation of the propargyl chain. In both molecules, the diazepine ring adopts a boat conformation with the propargyl-bearing C atom as the prow and the C atoms at the ring junction as the stern. The carbonyl O atom of one independent mol­ecule is hydrogen bonded to the acetyl­enic H atom of the other independent mol­ecule. In the crystal, symmetry-related mol­ecules are linked together by C—H⋯O hydrogen bonds, forming a ribbon-like structure along the *c* axis.

## Related literature

For a related structure, see: Jabli *et al.* (2009 ▶).
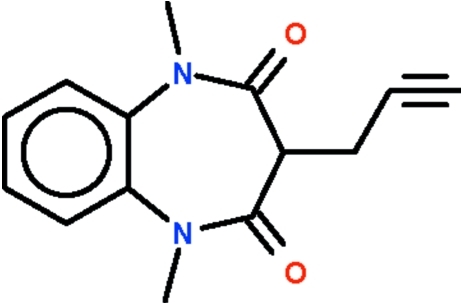


## Experimental

### 

#### Crystal data

C_14_H_14_N_2_O_2_
*M* *_r_* = 242.27
Monoclinic, 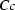

*a* = 16.0768 (3) Å
*b* = 17.1087 (3) Å
*c* = 8.9530 (2) Å
β = 93.701 (1)°
*V* = 2457.42 (8) Å^3^
*Z* = 8
Mo *K*α radiation
μ = 0.09 mm^−1^
*T* = 293 K
0.40 × 0.30 × 0.05 mm

#### Data collection

Bruker X8 APEXII area-detector diffractometer
21953 measured reflections
3580 independent reflections
2637 reflections with *I* > 2σ(*I*)
*R*_int_ = 0.049

#### Refinement

*R*[*F* ^2^ > 2σ(*F* ^2^)] = 0.038
*wR*(*F* ^2^) = 0.105
*S* = 1.06
3580 reflections
337 parameters
4 restraints
H atoms treated by a mixture of independent and constrained refinement
Δρ_max_ = 0.22 e Å^−3^
Δρ_min_ = −0.19 e Å^−3^

### 

Data collection: *APEX2* (Bruker, 2008 ▶); cell refinement: *SAINT* (Bruker, 2008 ▶); data reduction: *SAINT*; program(s) used to solve structure: *SHELXS97* (Sheldrick, 2008 ▶); program(s) used to refine structure: *SHELXL97* (Sheldrick, 2008 ▶); molecular graphics: *X-SEED* (Barbour, 2001 ▶); software used to prepare material for publication: *publCIF* (Westrip, 2010 ▶).

## Supplementary Material

Crystal structure: contains datablocks global, I. DOI: 10.1107/S1600536810024219/ci5110sup1.cif

Structure factors: contains datablocks I. DOI: 10.1107/S1600536810024219/ci5110Isup2.hkl

Additional supplementary materials:  crystallographic information; 3D view; checkCIF report

## Figures and Tables

**Table 1:** Hydrogen-bond geometry (Å, °)

*D*—H⋯*A*	*D*—H	H⋯*A*	*D*⋯*A*	*D*—H⋯*A*
C13—H13⋯O4	0.93 (3)	2.44 (3)	3.366 (3)	172 (3)
C8—H8⋯O2^i^	0.98	2.56	3.536 (3)	175
C19—H19⋯O1^i^	0.93	2.49	3.374 (3)	160

Symmetry code: (i) 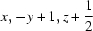.

**Fig. 1.**
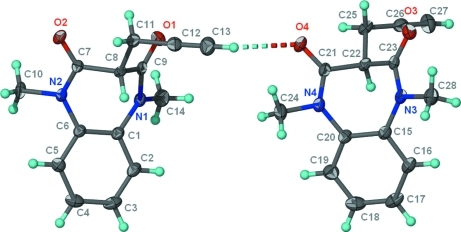
Thermal ellipsoid plot (Barbour, 2001) of the two independent molecules of C14H14N2O2 at the 50% probability level shown as a hydrogen-bonded dimer; H atoms are drawn as spheres of arbitrary radius. The dashed line denotes a hydrogen bond.

## supplementary crystallographic information

### Comment

We recently reported the crystal stucture of 1,5-dibenzyl-3-propargyl-1,5-benzodiazepine-2,4-dione, a compound readily synthesized by reacting the disubstituted 1,5-benzodiazepine-2,4-dione with propargyl bromide (Jabli *et al.*, 2009). The background to the study of such compounds is given in other reports. Replacing the benzyl unit by a methyl unit in the present study gives a compoound having a diazepine ring system (Scheme I, Fig. 1). This ring adopts a boat conformation (with the propargyl-bearing C atom as the prow and the fused-ring C atoms as the stern). There are two independent molecules; the acetylenic H-atom of one molecule forms a hydrogen to the carbonyl O-atom of the other independent molecule.

### Experimental

To a solution of potassium *t*-butoxide (0.42 g, 3.6 mmol) in DMF (15 ml) was added 1,5-dimethyl-1,5-benzodiazepine-2,4-dione (0.50 g, 2.4 mmol) and propargyl bromide (0.26 ml, 2.87 mmol). Stirring was continued for 24 h. The reaction was monitored by thin layer chromatography. On completion of the reaction, the mixture was filtered; crystals were obtained when the solvent was allowed to evaporate.

### Refinement

The acetylenic H atoms were located in a difference Fourier map and were refined with C–H distances restrained to 0.93 (1) Å; their U_iso_ parameters were freely refined. The remaining H atoms were placed in calculated positions (C–H = 0.93–0.98 Å) and were included in the refinement in the riding-model approximation, with *U_iso_*(H) set to 1.2–1.5*U*_eq_(C). In the absence of significant anomalous scattering effects, 3266 Friedel pairs were averaged.

### Crystal data

**Table:**

C_14_H_14_N_2_O_2_	*F*(000) = 1024
*M**_r_* = 242.27	*D*_x_ = 1.310 Mg m^−^^3^
Monoclinic, *C**c*	Mo *K*α radiation, λ = 0.71073 Å
Hall symbol: C -2yc	Cell parameters from 5448 reflections
*a* = 16.0768 (3) Å	θ = 2.5–29.4°
*b* = 17.1087 (3) Å	µ = 0.09 mm^−^^1^
*c* = 8.9530 (2) Å	*T* = 293 K
β = 93.701 (1)°	Plate, colourless
*V* = 2457.42 (8) Å^3^	0.40 × 0.30 × 0.05 mm
*Z* = 8	

### Data collection

**Table:**

Bruker X8 APEXII area-detector diffractometer	2637 reflections with *I* > 2σ(*I*)
Radiation source: fine-focus sealed tube	*R*_int_ = 0.049
graphite	θ_max_ = 30.0°, θ_min_ = 2.8°
φ and ω scans	*h* = −22→22
21953 measured reflections	*k* = −24→23
3580 independent reflections	*l* = −12→12

### Refinement

**Table:**

Refinement on *F*^2^	Primary atom site location: structure-invariant direct methods
Least-squares matrix: full	Secondary atom site location: difference Fourier map
*R*[*F*^2^ > 2σ(*F*^2^)] = 0.038	Hydrogen site location: inferred from neighbouring sites
*wR*(*F*^2^) = 0.105	H atoms treated by a mixture of independent and constrained refinement
*S* = 1.06	*w* = 1/[σ^2^(*F*_o_^2^) + (0.0514*P*)^2^ + 0.5357*P*] where *P* = (*F*_o_^2^ + 2*F*_c_^2^)/3
3580 reflections	(Δ/σ)_max_ = 0.001
337 parameters	Δρ_max_ = 0.22 e Å^−^^3^
4 restraints	Δρ_min_ = −0.19 e Å^−^^3^

### Fractional atomic coordinates and isotropic or equivalent isotropic displacement parameters (Å^2^)

**Table:**

	*x*	*y*	*z*	*U*_iso_*/*U*_eq_	
O1	0.50007 (11)	0.33109 (11)	0.5000 (2)	0.0339 (4)	
O2	0.34014 (11)	0.41769 (12)	0.21819 (19)	0.0332 (4)	
O3	0.99978 (11)	0.63153 (11)	0.9419 (2)	0.0363 (4)	
O4	0.78408 (12)	0.58439 (13)	0.74162 (19)	0.0380 (5)	
N1	0.39120 (12)	0.33841 (12)	0.6473 (2)	0.0268 (4)	
N2	0.26570 (12)	0.39080 (12)	0.4189 (2)	0.0253 (4)	
N3	0.91197 (13)	0.68624 (13)	1.1007 (2)	0.0293 (5)	
N4	0.75177 (12)	0.65404 (14)	0.9454 (2)	0.0298 (5)	
C1	0.31443 (15)	0.37280 (14)	0.6847 (3)	0.0251 (5)	
C2	0.29827 (17)	0.38043 (16)	0.8365 (3)	0.0318 (6)	
H2	0.3393	0.3670	0.9099	0.038*	
C3	0.22235 (19)	0.40758 (17)	0.8778 (3)	0.0382 (6)	
H3	0.2124	0.4121	0.9786	0.046*	
C4	0.16095 (18)	0.42805 (17)	0.7692 (3)	0.0384 (6)	
H4	0.1091	0.4449	0.7969	0.046*	
C5	0.17692 (16)	0.42341 (15)	0.6190 (3)	0.0308 (5)	
H5	0.1359	0.4385	0.5467	0.037*	
C6	0.25334 (15)	0.39661 (13)	0.5746 (3)	0.0240 (5)	
C7	0.33523 (15)	0.41709 (14)	0.3543 (3)	0.0242 (5)	
C8	0.40776 (14)	0.44186 (14)	0.4628 (2)	0.0232 (5)	
H8	0.3882	0.4782	0.5375	0.028*	
C9	0.43852 (14)	0.36629 (14)	0.5383 (3)	0.0250 (5)	
C10	0.19504 (16)	0.36807 (17)	0.3156 (3)	0.0329 (6)	
H10A	0.2141	0.3334	0.2408	0.049*	
H10B	0.1711	0.4139	0.2682	0.049*	
H10C	0.1537	0.3421	0.3704	0.049*	
C11	0.47672 (15)	0.48013 (16)	0.3774 (3)	0.0295 (5)	
H11A	0.4954	0.4434	0.3040	0.035*	
H11B	0.4540	0.5255	0.3239	0.035*	
C12	0.54896 (16)	0.50462 (16)	0.4769 (3)	0.0324 (5)	
C13	0.60798 (19)	0.5229 (2)	0.5539 (4)	0.0446 (7)	
H13	0.6549 (15)	0.537 (2)	0.614 (4)	0.062 (11)*	
C14	0.42070 (18)	0.26755 (16)	0.7274 (3)	0.0373 (6)	
H14A	0.4325	0.2277	0.6563	0.056*	
H14B	0.3784	0.2493	0.7898	0.056*	
H14C	0.4704	0.2794	0.7884	0.056*	
C15	0.83776 (15)	0.68259 (15)	1.1784 (3)	0.0279 (5)	
C16	0.84147 (18)	0.69589 (16)	1.3331 (3)	0.0343 (6)	
H16	0.8919	0.7095	1.3829	0.041*	
C17	0.7709 (2)	0.68902 (17)	1.4124 (3)	0.0404 (7)	
H17	0.7745	0.6968	1.5154	0.049*	
C18	0.6949 (2)	0.67062 (18)	1.3391 (3)	0.0429 (7)	
H18	0.6477	0.6658	1.3932	0.052*	
C19	0.68919 (17)	0.65948 (16)	1.1858 (3)	0.0357 (6)	
H19	0.6378	0.6481	1.1369	0.043*	
C20	0.75997 (16)	0.66527 (14)	1.1039 (3)	0.0278 (5)	
C21	0.79742 (15)	0.60039 (16)	0.8740 (3)	0.0281 (5)	
C22	0.87014 (15)	0.56424 (15)	0.9697 (3)	0.0274 (5)	
H22	0.8497	0.5453	1.0640	0.033*	
C23	0.93407 (15)	0.62914 (15)	1.0034 (3)	0.0272 (5)	
C24	0.67961 (17)	0.68895 (18)	0.8601 (3)	0.0386 (6)	
H24A	0.6957	0.7049	0.7634	0.058*	
H24B	0.6605	0.7336	0.9131	0.058*	
H24C	0.6356	0.6511	0.8484	0.058*	
C25	0.90761 (17)	0.49596 (16)	0.8873 (3)	0.0330 (6)	
H25A	0.8631	0.4621	0.8477	0.040*	
H25B	0.9365	0.5159	0.8034	0.040*	
C26	0.96610 (17)	0.45014 (16)	0.9847 (3)	0.0326 (6)	
C27	1.01106 (19)	0.41189 (19)	1.0637 (4)	0.0445 (7)	
H27	1.043 (2)	0.3804 (19)	1.130 (4)	0.070 (12)*	
C28	0.97458 (18)	0.74656 (18)	1.1443 (4)	0.0426 (7)	
H28A	1.0095	0.7556	1.0630	0.064*	
H28B	1.0082	0.7291	1.2305	0.064*	
H28C	0.9468	0.7942	1.1679	0.064*	

### Atomic displacement parameters (Å^2^)

**Table:**

	*U*^11^	*U*^22^	*U*^33^	*U*^12^	*U*^13^	*U*^23^
O1	0.0256 (9)	0.0397 (10)	0.0365 (11)	0.0051 (8)	0.0020 (7)	−0.0001 (8)
O2	0.0345 (9)	0.0472 (11)	0.0175 (9)	−0.0022 (8)	−0.0014 (7)	0.0014 (8)
O3	0.0284 (10)	0.0455 (11)	0.0350 (11)	−0.0010 (8)	0.0007 (8)	−0.0021 (9)
O4	0.0363 (10)	0.0558 (13)	0.0210 (10)	−0.0022 (9)	−0.0054 (8)	−0.0026 (8)
N1	0.0276 (10)	0.0311 (10)	0.0210 (10)	0.0018 (8)	−0.0029 (8)	0.0052 (9)
N2	0.0234 (10)	0.0323 (11)	0.0197 (10)	−0.0023 (8)	−0.0022 (8)	−0.0003 (8)
N3	0.0267 (10)	0.0350 (12)	0.0254 (11)	−0.0037 (8)	−0.0038 (8)	−0.0050 (9)
N4	0.0256 (10)	0.0398 (12)	0.0233 (11)	−0.0010 (8)	−0.0030 (8)	0.0021 (9)
C1	0.0275 (12)	0.0277 (12)	0.0202 (11)	−0.0040 (9)	0.0019 (9)	0.0009 (10)
C2	0.0372 (13)	0.0376 (14)	0.0204 (12)	−0.0069 (11)	0.0008 (10)	0.0013 (10)
C3	0.0507 (16)	0.0403 (15)	0.0249 (13)	−0.0065 (12)	0.0139 (12)	−0.0015 (12)
C4	0.0376 (15)	0.0383 (15)	0.0411 (16)	−0.0005 (12)	0.0161 (12)	−0.0007 (13)
C5	0.0272 (12)	0.0342 (13)	0.0312 (13)	−0.0017 (10)	0.0034 (10)	0.0002 (11)
C6	0.0256 (11)	0.0261 (11)	0.0202 (11)	−0.0039 (9)	0.0015 (9)	0.0003 (9)
C7	0.0244 (11)	0.0288 (12)	0.0194 (11)	0.0038 (9)	−0.0003 (8)	0.0019 (9)
C8	0.0243 (11)	0.0293 (12)	0.0162 (10)	−0.0010 (9)	0.0022 (8)	−0.0013 (9)
C9	0.0230 (11)	0.0309 (12)	0.0202 (11)	−0.0016 (9)	−0.0056 (9)	−0.0008 (9)
C10	0.0276 (13)	0.0400 (15)	0.0295 (14)	−0.0048 (11)	−0.0102 (10)	−0.0004 (11)
C11	0.0279 (12)	0.0380 (14)	0.0227 (12)	−0.0040 (10)	0.0017 (9)	0.0037 (10)
C12	0.0308 (13)	0.0374 (13)	0.0296 (13)	−0.0036 (11)	0.0060 (11)	0.0054 (11)
C13	0.0379 (15)	0.0542 (19)	0.0412 (17)	−0.0145 (14)	−0.0016 (13)	0.0030 (14)
C14	0.0414 (15)	0.0365 (15)	0.0335 (14)	0.0051 (12)	−0.0019 (11)	0.0121 (12)
C15	0.0316 (13)	0.0288 (13)	0.0231 (12)	0.0016 (10)	−0.0010 (10)	−0.0013 (10)
C16	0.0438 (15)	0.0345 (14)	0.0238 (13)	0.0031 (11)	−0.0047 (11)	−0.0013 (11)
C17	0.0598 (18)	0.0374 (15)	0.0244 (13)	0.0026 (13)	0.0047 (13)	0.0006 (12)
C18	0.0497 (17)	0.0412 (17)	0.0401 (17)	−0.0037 (13)	0.0199 (14)	0.0010 (13)
C19	0.0337 (14)	0.0384 (15)	0.0356 (15)	−0.0055 (11)	0.0056 (12)	−0.0004 (12)
C20	0.0317 (12)	0.0284 (12)	0.0233 (12)	−0.0028 (10)	0.0011 (10)	0.0004 (10)
C21	0.0254 (11)	0.0374 (13)	0.0210 (12)	−0.0078 (10)	−0.0016 (9)	0.0018 (10)
C22	0.0312 (12)	0.0335 (13)	0.0170 (11)	−0.0003 (10)	−0.0016 (9)	0.0007 (10)
C23	0.0250 (11)	0.0353 (13)	0.0202 (12)	0.0019 (9)	−0.0056 (9)	0.0023 (10)
C24	0.0291 (13)	0.0506 (17)	0.0351 (15)	0.0009 (12)	−0.0066 (11)	0.0030 (13)
C25	0.0422 (14)	0.0367 (14)	0.0197 (12)	−0.0012 (11)	−0.0007 (11)	−0.0029 (10)
C26	0.0341 (13)	0.0362 (14)	0.0279 (13)	−0.0034 (11)	0.0051 (10)	−0.0044 (11)
C27	0.0344 (15)	0.0495 (18)	0.0488 (19)	−0.0012 (13)	−0.0036 (13)	0.0078 (14)
C28	0.0351 (14)	0.0497 (17)	0.0423 (17)	−0.0121 (13)	−0.0030 (12)	−0.0113 (14)

### Geometric parameters (Å, °)

**Table:**

O1—C9	1.226 (3)	C11—C12	1.478 (4)
O2—C7	1.226 (3)	C11—H11A	0.97
O3—C23	1.223 (3)	C11—H11B	0.97
O4—C21	1.222 (3)	C12—C13	1.179 (4)
N1—C9	1.361 (3)	C13—H13	0.93 (3)
N1—C1	1.427 (3)	C14—H14A	0.96
N1—C14	1.472 (3)	C14—H14B	0.96
N2—C7	1.367 (3)	C14—H14C	0.96
N2—C6	1.424 (3)	C15—C16	1.401 (3)
N2—C10	1.470 (3)	C15—C20	1.411 (3)
N3—C23	1.371 (3)	C16—C17	1.381 (4)
N3—C15	1.421 (3)	C16—H16	0.93
N3—C28	1.476 (3)	C17—C18	1.385 (4)
N4—C21	1.360 (3)	C17—H17	0.93
N4—C20	1.430 (3)	C18—C19	1.383 (4)
N4—C24	1.473 (3)	C18—H18	0.93
C1—C6	1.406 (3)	C19—C20	1.397 (4)
C1—C2	1.406 (4)	C19—H19	0.93
C2—C3	1.379 (4)	C21—C22	1.535 (3)
C2—H2	0.93	C22—C25	1.526 (4)
C3—C4	1.385 (4)	C22—C23	1.530 (3)
C3—H3	0.93	C22—H22	0.98
C4—C5	1.388 (4)	C24—H24A	0.96
C4—H4	0.93	C24—H24B	0.96
C5—C6	1.393 (4)	C24—H24C	0.96
C5—H5	0.93	C25—C26	1.468 (4)
C7—C8	1.528 (3)	C25—H25A	0.97
C8—C9	1.526 (3)	C25—H25B	0.97
C8—C11	1.534 (3)	C26—C27	1.177 (4)
C8—H8	0.98	C27—H27	0.93 (3)
C10—H10A	0.96	C28—H28A	0.96
C10—H10B	0.96	C28—H28B	0.96
C10—H10C	0.96	C28—H28C	0.96
			
C9—N1—C1	123.77 (19)	N1—C14—H14A	109.5
C9—N1—C14	117.4 (2)	N1—C14—H14B	109.5
C1—N1—C14	118.8 (2)	H14A—C14—H14B	109.5
C7—N2—C6	124.09 (19)	N1—C14—H14C	109.5
C7—N2—C10	116.2 (2)	H14A—C14—H14C	109.5
C6—N2—C10	118.88 (19)	H14B—C14—H14C	109.5
C23—N3—C15	122.7 (2)	C16—C15—C20	118.8 (2)
C23—N3—C28	117.8 (2)	C16—C15—N3	119.6 (2)
C15—N3—C28	118.7 (2)	C20—C15—N3	121.6 (2)
C21—N4—C20	122.6 (2)	C17—C16—C15	120.7 (2)
C21—N4—C24	117.4 (2)	C17—C16—H16	119.7
C20—N4—C24	118.6 (2)	C15—C16—H16	119.7
C6—C1—C2	119.1 (2)	C16—C17—C18	120.3 (3)
C6—C1—N1	122.0 (2)	C16—C17—H17	119.9
C2—C1—N1	118.8 (2)	C18—C17—H17	119.9
C3—C2—C1	120.8 (2)	C17—C18—C19	120.1 (3)
C3—C2—H2	119.6	C17—C18—H18	120.0
C1—C2—H2	119.6	C19—C18—H18	120.0
C2—C3—C4	120.0 (3)	C18—C19—C20	120.5 (3)
C2—C3—H3	120.0	C18—C19—H19	119.8
C4—C3—H3	120.0	C20—C19—H19	119.8
C3—C4—C5	119.8 (3)	C19—C20—C15	119.6 (2)
C3—C4—H4	120.1	C19—C20—N4	119.1 (2)
C5—C4—H4	120.1	C15—C20—N4	121.3 (2)
C4—C5—C6	121.1 (3)	O4—C21—N4	122.7 (2)
C4—C5—H5	119.4	O4—C21—C22	122.1 (2)
C6—C5—H5	119.4	N4—C21—C22	115.1 (2)
C5—C6—C1	119.0 (2)	C25—C22—C23	111.7 (2)
C5—C6—N2	118.9 (2)	C25—C22—C21	110.40 (19)
C1—C6—N2	122.1 (2)	C23—C22—C21	107.2 (2)
O2—C7—N2	122.0 (2)	C25—C22—H22	109.2
O2—C7—C8	122.3 (2)	C23—C22—H22	109.2
N2—C7—C8	115.7 (2)	C21—C22—H22	109.2
C9—C8—C7	105.00 (19)	O3—C23—N3	121.9 (2)
C9—C8—C11	111.0 (2)	O3—C23—C22	121.6 (2)
C7—C8—C11	110.36 (19)	N3—C23—C22	116.4 (2)
C9—C8—H8	110.1	N4—C24—H24A	109.5
C7—C8—H8	110.1	N4—C24—H24B	109.5
C11—C8—H8	110.1	H24A—C24—H24B	109.5
O1—C9—N1	121.8 (2)	N4—C24—H24C	109.5
O1—C9—C8	122.4 (2)	H24A—C24—H24C	109.5
N1—C9—C8	115.7 (2)	H24B—C24—H24C	109.5
N2—C10—H10A	109.5	C26—C25—C22	112.3 (2)
N2—C10—H10B	109.5	C26—C25—H25A	109.1
H10A—C10—H10B	109.5	C22—C25—H25A	109.1
N2—C10—H10C	109.5	C26—C25—H25B	109.1
H10A—C10—H10C	109.5	C22—C25—H25B	109.1
H10B—C10—H10C	109.5	H25A—C25—H25B	107.9
C12—C11—C8	112.7 (2)	C27—C26—C25	178.0 (3)
C12—C11—H11A	109.1	C26—C27—H27	176 (3)
C8—C11—H11A	109.1	N3—C28—H28A	109.5
C12—C11—H11B	109.1	N3—C28—H28B	109.5
C8—C11—H11B	109.1	H28A—C28—H28B	109.5
H11A—C11—H11B	107.8	N3—C28—H28C	109.5
C13—C12—C11	178.1 (3)	H28A—C28—H28C	109.5
C12—C13—H13	180 (3)	H28B—C28—H28C	109.5
			
C9—N1—C1—C6	44.7 (3)	C23—N3—C15—C16	131.1 (3)
C14—N1—C1—C6	−133.7 (3)	C28—N3—C15—C16	−38.5 (3)
C9—N1—C1—C2	−137.7 (2)	C23—N3—C15—C20	−48.5 (3)
C14—N1—C1—C2	43.9 (3)	C28—N3—C15—C20	142.0 (3)
C6—C1—C2—C3	2.9 (4)	C20—C15—C16—C17	2.7 (4)
N1—C1—C2—C3	−174.9 (2)	N3—C15—C16—C17	−176.9 (3)
C1—C2—C3—C4	−0.3 (4)	C15—C16—C17—C18	−1.5 (4)
C2—C3—C4—C5	−1.9 (4)	C16—C17—C18—C19	−0.4 (4)
C3—C4—C5—C6	1.6 (4)	C17—C18—C19—C20	1.1 (4)
C4—C5—C6—C1	1.0 (4)	C18—C19—C20—C15	0.0 (4)
C4—C5—C6—N2	178.2 (2)	C18—C19—C20—N4	−179.5 (3)
C2—C1—C6—C5	−3.2 (3)	C16—C15—C20—C19	−1.9 (4)
N1—C1—C6—C5	174.4 (2)	N3—C15—C20—C19	177.7 (2)
C2—C1—C6—N2	179.8 (2)	C16—C15—C20—N4	177.6 (2)
N1—C1—C6—N2	−2.6 (3)	N3—C15—C20—N4	−2.8 (4)
C7—N2—C6—C5	133.8 (2)	C21—N4—C20—C19	−124.8 (3)
C10—N2—C6—C5	−35.1 (3)	C24—N4—C20—C19	41.7 (3)
C7—N2—C6—C1	−49.2 (3)	C21—N4—C20—C15	55.7 (3)
C10—N2—C6—C1	141.9 (2)	C24—N4—C20—C15	−137.8 (2)
C6—N2—C7—O2	−173.0 (2)	C20—N4—C21—O4	171.5 (2)
C10—N2—C7—O2	−3.8 (3)	C24—N4—C21—O4	4.8 (4)
C6—N2—C7—C8	9.9 (3)	C20—N4—C21—C22	−11.6 (3)
C10—N2—C7—C8	179.0 (2)	C24—N4—C21—C22	−178.2 (2)
O2—C7—C8—C9	−109.3 (3)	O4—C21—C22—C25	−12.3 (3)
N2—C7—C8—C9	67.9 (3)	N4—C21—C22—C25	170.7 (2)
O2—C7—C8—C11	10.4 (3)	O4—C21—C22—C23	109.5 (3)
N2—C7—C8—C11	−172.4 (2)	N4—C21—C22—C23	−67.4 (3)
C1—N1—C9—O1	−173.1 (2)	C15—N3—C23—O3	−177.0 (2)
C14—N1—C9—O1	5.3 (3)	C28—N3—C23—O3	−7.4 (4)
C1—N1—C9—C8	4.2 (3)	C15—N3—C23—C22	6.0 (3)
C14—N1—C9—C8	−177.4 (2)	C28—N3—C23—C22	175.6 (2)
C7—C8—C9—O1	100.1 (2)	C25—C22—C23—O3	14.7 (3)
C11—C8—C9—O1	−19.1 (3)	C21—C22—C23—O3	−106.3 (3)
C7—C8—C9—N1	−77.2 (2)	C25—C22—C23—N3	−168.3 (2)
C11—C8—C9—N1	163.55 (19)	C21—C22—C23—N3	70.7 (3)
C9—C8—C11—C12	−63.5 (3)	C23—C22—C25—C26	72.0 (3)
C7—C8—C11—C12	−179.5 (2)	C21—C22—C25—C26	−168.9 (2)

### Hydrogen-bond geometry (Å, °)

**Table:**

*D*—H···*A*	*D*—H	H···*A*	*D*···*A*	*D*—H···*A*
C13—H13···O4	0.93 (3)	2.44 (3)	3.366 (3)	172 (3)
C8—H8···O2^i^	0.98	2.56	3.536 (3)	175
C19—H19···O1^i^	0.93	2.49	3.374 (3)	160

Symmetry codes: (i) *x*, −*y*+1, *z*+1/2.
